# Amplification of resonant field enhancement by plasmonic lattice coupling in metallic slit arrays

**DOI:** 10.1038/srep37738

**Published:** 2016-11-25

**Authors:** Pernille Klarskov, Abebe T. Tarekegne, Krzysztof Iwaszczuk, X.-C. Zhang, Peter Uhd Jepsen

**Affiliations:** 1 DTU Fotonik - Department of Photonics Engineering, Technical University of Denmark, DK-2800 Kongens Lyngby, Denmark; 2 Institute of Optics, University of Rochester, Rochester, NY 14627-0186, United States of America

## Abstract

Nonlinear spectroscopic investigation in the terahertz (THz) range requires significant field strength of the light fields. It is still a challenge to obtain the required field strengths in free space from table-top laser systems at sufficiently high repetition rates to enable quantitative nonlinear spectroscopy. It is well known that local enhancement of the THz field can be obtained for instance in narrow apertures in metallic films. Here we show by simulation, analytical modelling and experiment that the achievable field enhancement in a two-dimensional array of slits with micrometer dimensions in a metallic film can be increased by at least 60% compared to the enhancement in an isolated slit. The additional enhancement is obtained by optimized plasmonic coupling between the lattice modes and the resonance of the individual slits. Our results indicate a viable route to sensitive schemes for THz spectroscopy with slit arrays manufactured by standard UV photolithography, with local field strengths in the multi-ten-MV/cm range at kHz repetition rates, and tens of kV/cm at oscillator repetition rates.

The established THz technology has proven its relevance for applications within spectroscopy and imaging by providing fundamental information about chemical composition, conductivity or composition of hidden layers^1^,^2^. With the recent advances of table-top THz systems offering intense ultrashort pulses with field strengths well in the MV/cm regime^3^,^4^, the number of studies of nonlinear effects in the THz range is steadily growing^5^,^6^,^7^, where the main focus until now has been on semiconductor systems with a strong electronic response. Still, insufficiently high field strengths seem to be the limiting factor for nonlinear studies of materials with a weaker nonlinear response, such as molecular crystals, where only a few results on the nonlinear response has been reported^5^. Recently, nanoslits were applied for THz spectroscopy studies of crystalline materials since the electric field as well as the absorption cross-section can be enhanced dramatically inside the slit^8^. Combining such field-enhancing structures with an intense THz source a field enhancement to 20 MV/cm has been achieved^9^. The linear properties of phonon modes of semiconductor quantum dots have been studied with the dots placed in a nanogap between closely spaced metallic antennas with strong, resonant field enhancement of the incoming THz field^10^, and linear optical properties of monolayers of proteins in the field-enhanced region near nano-antennas have been studied in the mid-infrared^11^.

Transmission through subwavelength apertures has attracted a lot of attention since Bethe’s demonstration of transmission through a subwavelength hole in 1940’s^12^. Later, the field enhancement inside metallic apertures such as nano-holes^13^, rectangular apertures^14^ and slits^15^ has been studied extensively with emphasis on the extraordinary transmission (EOT) of light through such structures. It has been shown that field enhancements of tens of thousands can be achieved if the gap size is on the order of a nm for THz waves of low field strength^16^. Nano-gap structures in metal surfaces have in addition been utilized to demonstrate THz induced electron tunneling across the gap^17^. The fabrication of such nano-structures requires advanced techniques such as e-beam lithography^10^, focused ion beams (FIB)^18^ or even atomic layer lithography^16^. While other more standard methods such as UV photolithography do not offer the nanometer resolution required to fabricate these extreme field-enhancing structures, UV lithography is orders of magnitude less costly, and hence easier to access. At the same time having a parallelized lithography technique based on a simple mask design allows for low-cost mass production of an ideal structure design.

Lattice modes in a periodic array of resonant structures can couple to the resonance of the structures itself^19^,^20^. In particular, the interplay between aperture resonances and lattice modes has been studied in the microwave domain where the metal conductivity is high and the large-scale wavelength-determined features offers uncomplicated fabrication methods^21^, but the transmission through hole arrays is today well described at all wavelengths in the electromagnetic spectrum^22^. While the nature of the coupling between apertures arranged in a periodic array in a metallic film is of plasmonic nature^23^, comparably sparse information and design criteria on how to exploit this coupling for additional field enhancement inside the apertures is available in the literature. Yet, tailoring of this coupling to achieve the highest possible field enhancement is relevant for spectroscopy systems in the THz range. Due to the high conductivity of metals in the THz range, surface plasmon polaritons (SPPs) are weakly confined to the metal interface in the THz range. Coupling of the electromagnetic field to metal surfaces in the THz range is well-established^24^, with several demonstrations of plasmonic localization of THz waves^25^,^26^,^27^,^28^.

Here we demonstrate how rectangular slit apertures (also called slot antennas) with transmission resonance frequencies in the low THz range, arranged periodically in a metallic film, can be designed so that the lattice modes constructively interfere with the slit resonance leading to a field enhancement inside the slit which is up to 60% larger than of an isolated slit of the same dimensions. With apertures fabricated by standard UV photolithography we demonstrate a field enhancement of up to approximately 40 times in the center of the aperture while maintaining an overall transmission through the array of up to 10%, thus enabling sensitive detection of the transmitted field. We show that this additional enhancement is due to resonant coupling mediated by standing SPP waves between the the microslit resonators. The coupling behavior between microslits and lattice modes is reproduced by an analytical model of coupled harmonic oscillators. We emphasize that the aim of the present investigation is to shed light on this very general amplification mechanism of the resonant field enhancement, and not on the absolute magnitude of the enhancement itself, which as discussed above can be orders of magnitude larger in nanometer-wide slits.

## Theoretical calculations of resonance frequencies

EOT occurs when the impedance is matched through a resonant aperture^29^,^30^. The resonant length of a half-wavelength dipole antenna is defined as the length where the reactance (imaginary part of the impedance *Z*_*in*_ = *R*_*in*_ + *iX*_*in*_) is zero. This length is slightly shorter than *λ*_0_/2^31^, where *λ*_0_ = *c/v*_0_ is the resonance wavelength in free space, and *v*_0_ is the corresponding frequency. A redshift of the resonant transmission wavelength through rectangular apertures has been observed both in simulations assuming that the metal is a perfect electric conductor^32^ and experiments at near-infrared and visible wavelengths^33^ where the conductivity of metals is significantly lower than in the THz range. For electric fields polarized perpendicular to the long axis of a slit aperture a buildup of opposite charges along the slit walls is induced by the incoming electric field. At resonance the currents at the end points of the slit aperture vanish, resulting in charge accumulation at each side of the aperture and consequently strong field enhancement. When a slot antenna is placed on a semi-infinite substrate, the optimal length *L* for a given width *w*, and resonance frequency *v*_0_ of the antenna is given the same expression as for a thin wire antenna, modified by a factor determined by the permittivity of the substrate^31^,^34^,^35^,


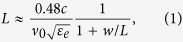


where *c* is the speed of light in vacuum, *ε*_*e*_ = (1 + *n*^2^)/2 is the effective permittivity of a semi-infinite substrate, and *n* is the refractive index of the substrate material (*n* = 3.4177 for silicon^36^). Park *et al.*^18^ have shown this approximation to be valid for SiN substrates thicker than 60 μm, and here we consider Si substrates with a thickness of 525 μm.

**Figure 1:**
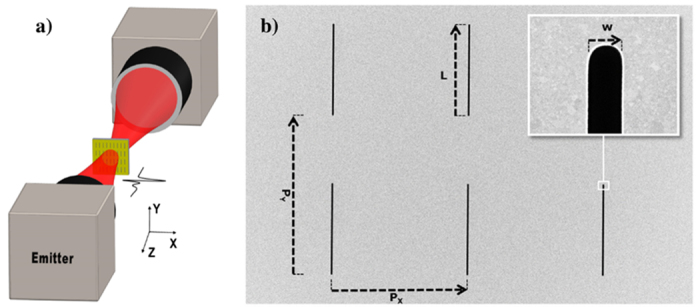
(**a**) Experimental configuration of a slit array measured with a THz-TDS system. (**b**) SEM image of a typical slit array. Then inset shows a zoom of a slit. In this specific case, *L* = 50 μm, *P*_*x*_ = 70 μm, *P*_*y*_ = 110 μm, and *w* = 1.5 μm.

The enhancement of an incoming electric field at the resonance frequency in an isolated, sub-wavelength (*L*, *w* < *λ*_0_) slit aperture in an infinite metallic ground plane can be described analytically^32^. In a periodic array of holes in a thin metal film the transmission is influenced by coupling between the field transmitted through the individual apertures and SPP-mediated lattice modes. In this situation, EOT can be observed both at the slit aperture resonance and at the additional lattice mode resonances^23^. In the THz range where the conductivity of metals is very high and the SPP propagation constant consequently is close to that of free space, the lattice mode frequencies are determined by a standing-wave condition similar to Wood anomalies^37^


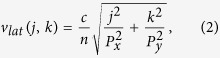


where *j* and *k* are integers, *P*_*x*_ and *P*_*y*_ are the lattice constants (see Fig. 1), and *n* is the refractive index of the material above the metal film.

Equation (2) assumes normal incidence while angular dependent measurements of THz resonant structures have been reported showing a strong angular dependence when a Fano-shaped lattice mode is coupled to the structural resonance^38^. However, the maximum transmission was found be at normal incidence excitation, which is desired for this study. The lattice modes can be excited on both the air-metal and metal-substrate interfaces. Here we consider metal films thicker than the skin depth, and thus SPP waves on each side of the metal film are independent of each other. Because of the higher refractive index, the frequency-dependent transmission of the field will be mainly influenced by the SPP waves on the metal-substrate interface. Bitzer *et al.*^20^ showed that at resonance, the spatial radiation pattern from an array of dipoles develops strong side lobes in the plane of the interface, giving rise to significant electromagnetic coupling between adjacent elements. In a transmission experiment with resonant elements periodically spaced on a dielectric substrate, these lattice resonances lead to losses in the transmitted far field. In the case of apertures in a metallic film, the component of the field that couples to SPP modes along the metal interface can interfere constructively with the enhanced field in the neighboring apertures. This offers the possibility of additional field enhancement in the apertures of the array and thus to further increase of the extraordinary transmission through the array^19^ compared to that in an isolated aperture where the SPP-coupled energy is lost.

## Field enhancement in periodically arranged slits

The effect of the lattice modes on the field enhancement inside slits has been studied in three cases where the slit length *L* and lattice parameters *P*_*x*_ and *P*_*y*_ (see Fig. 1(b)) have been varied individually. The exact parameters of the samples can be found in Table 1. The field enhancements are experimentally measured with a standard THz-TDS setup (see Fig. 1(a)), while full-wave electromagnetic simulations have been performed using a probe in the center of a slit.

**Figure 2:**
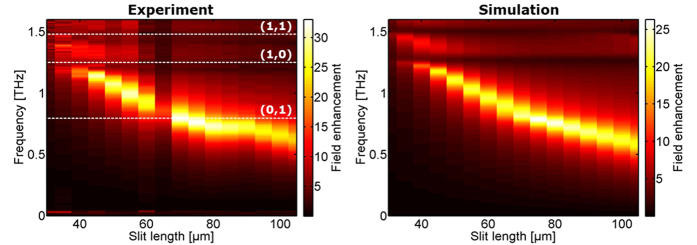
Experimental (left) and simulation (right) results of field enhancement inside slits when the length (*L*) is varied and the lattice parameters are *P*_*x*_ = 70 μm and *P*_*y*_ = 110 μm. The white dashed lines indicate lattice modes.

**Figure 3:**
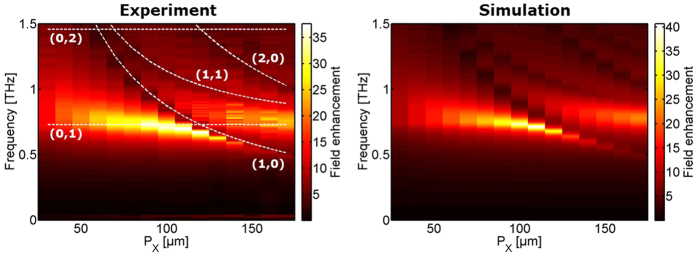
Experimental (left) and simulation (right) results of field enhancement inside slits when *P*_*x*_ is varied while *L* = 75** **μm and *P*_*y*_ = 120** **μm.

**Figure 4:**
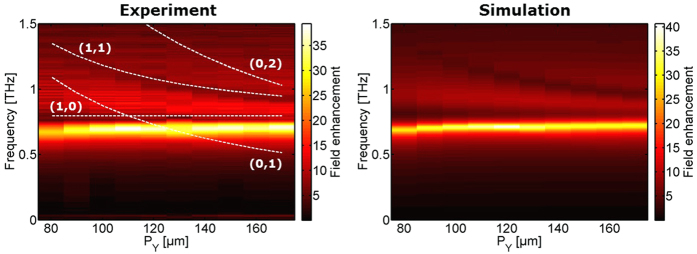
Experimental (left) and simulation (right) results of field enhancement inside slits when *P*_*y*_ is varied while *L* = 75 μm and *P*_*x*_ = 110 μm.

The results of the measured field enhancement are shown in the left columns of the Figs 2, 3 and 4, where the right columns are corresponding simulations. The calculated lattice modes (Equation 2) are indicated by white dashed lines showing the order (*j*, *k*).

Figure 2 shows the results from sample series I where *L* is tuned from 30 to 105 μm in steps of 5 μm. When *L* is increased the resonance frequency is reduced as expected. The field enhancement is highest when the slit resonance crosses the lattice mode resonance (0,1) whereas the lattice modes (1,0) and (1,1) result in dark bands of destructive interference, typical for Fano resonances in plasmonic structures^39^. This behavior is observed for coupling to lattice modes having a non-zero mode order in the horizontal direction (*j* ≠ 0, *k*), as expected due to the polarization of the THz field. However, there is still coupling to other lattice modes, leading to additional enhancement of the field as the slit resonance crosses the (0,1) lattice mode. Although the (0,1) lattice mode itself is not visible in neither experiment nor simulation, the effect on the field enhancement is obvious. For the experiment where *L* = 65 μm a significant lower transmission (and thus lower field enhancement) than the neighboring samples is observed, although the resonance is at the expected frequency. We inspected this particular sample with an optical microscope, and found no visible damage to the surface or unexpected deviations from the design dimensions, so the reason for the isolated discrepancy is not clear.

The coupling to the lattice modes in the horizontal direction is investigated with the sample series II where *L* and *P*_*y*_ are fixed, and *P*_*x*_ is varied so that the lowest order lattice mode resonance (1,0) crosses the slit resonance. The results of the experiments and simulations when varying *P*_*x*_ are shown in Fig. 3. The calculated lattice modes that only have a component in the y-direction, i.e. of the orders (0, *k*), are indicated with horizontal dashed lines, whereas the frequencies of the calculated lattice modes (*j* ≠ 0, *k*) are reduced when *P*_*x*_ is increased. These lattice modes are again clearly visible in the measurements and simulations as dark bands due to the Fano-like behavior, and agreeing well with the calculated lattice mode frequencies. Most significant is the (1,0) mode. When this approaches the slit resonance, the slit resonance redshifts slightly and sharpens. When the lattice mode crosses the slit resonance, it blueshifts rapidly as expected for an avoided crossing due to the coupling between the two modes. The abruptly increased damping after the crossing (*P*_*x*_ > *c/v*_*lat*_(1,0)) is due to radiative loss as the array starts to behave as a diffraction grating^38^. The field enhancement of the slit resonance reaches its highest value of more than 35 just before this avoided crossing. The (0,1) lattice mode is again not visible, but serves to further strengthen the field enhancement with the avoided crossing between the slit mode and the (1,0) lattice mode pushes the slit mode into resonance with the (0,1) mode.

The results of the series III of samples where *P*_*y*_ is varied are shown in Fig. 4. The fixed value of *P*_*x*_ is chosen so that the (0,1) lattice mode is slightly blueshifted from the slit resonance. Even though no spectral signatures from the lattice modes (0,1) and (0,2) are seen, we observe again an additional enhancement when the frequency of the lattice mode (0,1) approaches the slit resonance, similar to the behavior seen in sample series I and II.

## Quantitative field enhancement comparison

**Figure 5:**
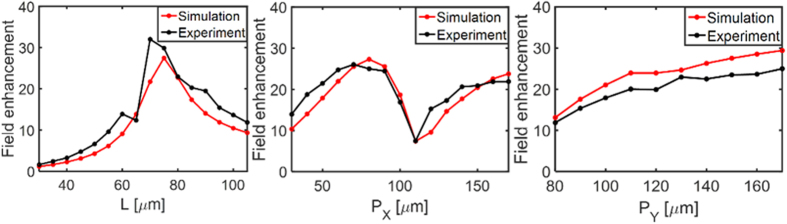
Averaged field enhancement obtained a slit (z = 0) at 0.79 THz by simulation (black) and experiment (red) when (**a**) *L* is varied while *P*_*x*_ = 70 μm and *P*_*y*_ = 110 μm, (**b**) *P*_*x*_ is varied while *P*_*y*_ = 120 μm and *L* = 75 μm. (**c**) Averaged field enhancement at 0.74 THz when *P*_*y*_ is varied while *P*_*x*_ = 110 μm and *L* = 75 μm.

The spectral features of resonances in experiments versus simulations for the three series of samples shown in Figs 2, 3 and 4 are all in good agreement. The measured field enhancements represent an averaged value over the slit area while the simulations are obtained with a probe in the center of the slit (x = y = z = 0). However, as it will be shown in the following the field distribution inside the slit is not uniform, and the experimentally measured field enhancement cannot be directly compared to the simulated probe-value quantitatively. For a quantitative comparison, Fig. 5 shows the experimentally obtained field enhancement at a specific frequency together with the simulated field enhancement at this frequency averaged over the slit area (at z = 0). For the series I and II where *L* and *P*_*x*_ are varied, respectively, the field enhancement is compared at the (0,1) lattice mode at 0.79 THz (Fig. 5(a) and (b)). In Fig. 5(c) where *P*_*y*_ is varied the field enhancement is compared at 0.74 THz, which is the theoretical value of the slit resonance calculated with Eq. 1.

In general, the experimentally observed and simulated field enhancements are in reasonably good quantitative agreement, both concerning the magnitude and the behavior when the different dimensions are varied. We observe additional enhancement when the lattice modes match the slit resonance, both with variation of *L*, *P*_*x*_ and *P*_*y*_. This is in contrast to the expected enhancement for an isolated slit which should increase monotonically with *L*, and demonstrates the positive effect of coupling between the lattice modes and the slit resonances on the obtainable field enhancement.

## Field distributions

**Figure 6:**
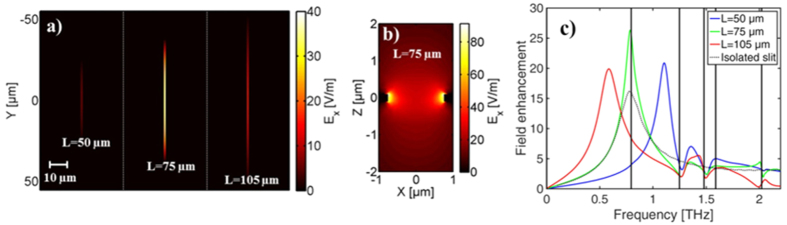
(**a**) Field distribution of E_x_ shown in the xy-plane (z = 0) inside three slits of lengths *L* = 50, 75 and 105 μm, with *P*_*x*_ = 70 μm and *P*_*y*_ = 110 μm. (**b**) E_x_ shown in the xz-plane (y = 0) on resonance for *L* = 75 μm. (**c**) Spectral distribution of the field enhancement probed in the center of the slits. The dotted line shows the field enhancement for an isolates slit with *L* = 75 μm and vertical solid lines indicate lattice modes.

Figure 6(a) shows the distribution of field enhancement in slits of three different lengths each in an array of identical slits with lattice pitch *P*_*x*_ = 70 μm and *P*_*y*_ = 110 μm at 0.79 THz, i.e. the frequency of the (0,1) lattice mode. It is seen that the field distribution is similar to the TE_01_ waveguide mode, with the highest field strength at *L*/2. The field enhancement is largest for *L* = 75 μm, where the slit resonance overlaps well with the lattice mode. A view of the field distribution of *E*_*x*_ in the xz-plane (y = 0) shows that the field is highest close to the edges where the field enhancement reaches a value of more than 80, twice as large as at the center (Fig. 6(b)). Here the gold layer is centered z = 0 with a thickness of 0.2 μm, and the silicon substrate is located at z > 0.1 μm. The spectral distributions of the field enhancement in the center of the three slits are shown in Fig. 6(c) where the coupling of the slit resonance to the lattice modes again is clearly seen. For comparison, the field enhancement in an isolated slit of *L* = 75 μm is indicated by the dashed line, showing a 40% lower field enhancement than in an array where the slit resonance is coupled to the (0,1) lattice mode.

**Figure 7:**
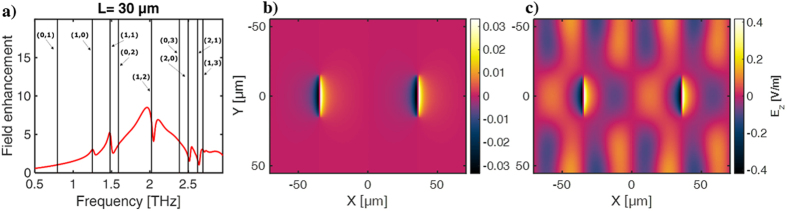
Field distribution (E_z_) of lattice modes in slit arrays of *L* = 30, *P*_*x*_ = 70 μm and *P*_*y*_ = 110 μm probed in the silicon substrate at z = 0.1 μm above the metal at the frequencies (**a**) *v*_*lat*_(0,1) = 0.79 THz and (**b**) *v*_*lat*_(2,1) = 2.61 THz.

To investigate the spatial distribution of the lattice modes, which on a metallic film are expected to be SPP modes, simulations of the z-component of the field (*E*_*z*_) are performed. Figure 7(b) and (c) show the field distribution of *E*_*z*_ in the xy-plane on the backside of the gold surface, 0.5 μm into the substrate. To enable excitation of higher-order lattice modes, the slit length is here chosen to be *L* = 30 μm, while the lattice size is again 70 × 110 μm. For this case, the field enhancement obtained with a probe in the center of a slit with full-wave simulation is shown as the red curve in Fig. 7(a), and all lattice modes in this frequency range are indicated as black lines. The field distribution at the lowest lattice mode (0,1) is shown in Fig. 7(b) where it is seen that the field is concentrated near the slits (located at x = ±35 μm, respectively). The field pattern at the lattice mode (2,1) is shown in Fig. 7(c) where the standing SPP waves between the neighboring slits are clearly seen. From the field values in (b) and (c) it is noted that the coupling to the (2,1) mode is significantly higher than to the (0,1). As it also will be discussed in our analytical model it is in general expected that the coupling is much higher to the modes with a non-zero component in the x-direction i.e. (*j* ≠ 0, *k*) and in addition the resonance frequency of the slit is in this case closer to the (2,1) lattice mode than the (0,1) mode. In Fig. 7(a) it is again noted that the (*j* ≠ 0, *k*) modes give rise to a Fano resonance in contrast to the (*j* = 0, *k*) modes. The standing waves observed between the neighboring slits consequently entails that at the resonance frequencies of the lattice modes the energy flow in and out of the slits balances.

## Simulation of coupling mechanism between slit and lattice modes

**Figure 8:**
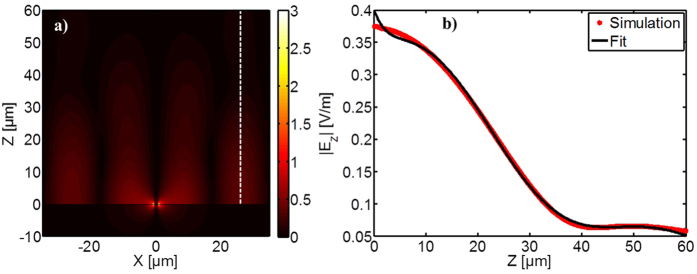
z component of the electric field (|E_z_|) simulated at *v*_*lat*_(2,1) = 2.61 THz for slit arrays of *L* = 30 μm, *P*_*x*_ = 70 μm and *P*_*y*_ = 110 μm in (**a**) the xz-plane and (**b**) along z at x = 26** **μm (red curve, marked with the dashed line in (**a**)). The black curve is a fit to the simulated data based on a superposition of a spherical wave and a SPP wave.

To study how the electric field decays from metallic surface compared to plasmonic waves, the z-component in the xz-plane is investigated. In Fig. 8 we show |*E*_*z*_| in the xz-plane for *v*_*lat*_(2,1) = 2.61 THz after plane-wave excitation from the air side. The slit is located at (0,0) (air side: z < 0, Si side: z > 0). The field pattern of standing waves observed in Fig. 7(b) is present again in Fig. 8(a), and it is observed that the field is concentrated in the Si substrate. A cross section at the second local maximum in the substrate at x = 26 μm (dashed line in Fig. 8(a)) is plotted as the red curve in Fig. 8(b). A simple superposition of a spherical wave and a standing SPP wave (exponentially decaying in the z-direction), shown as the black curve, reproduces most of the features. The SPP penetration depth into the substrate is substantial (tens of μm) due to the high conductivity of the metal. Nevertheless, the characteristics of an SPP wave are clearly present, indicating that the coupling between slits is indeed due to SPP waves, and not unbound waves which form the coupling mechanism between resonant elements supported by a dielectric substrate^20^. The amplitudes for the spherical wave and the SPP wave in the fit suggest that the coupling of the field in the slit to the spherical wave is approximately twice as strong as the coupling to the SPP wave. This ratio is indicative of the substantial effect of the plasmonic coupling between the slits in the array, and comparable to the observed amplification of the enhancement in the slits arranged periodically in an array compared to the enhancement in an isolated slit. In the work by Cao and Lalanne it was similarly shown that the transmitted field was influenced both by plasmonic and diffraction effects^40^. Here we clearly achieve a positive effect from excitation of SPPs when these are matched to the resonance of the slit mode.

## Analytical model of the interaction between slit and lattice resonances

**Figure 9:**
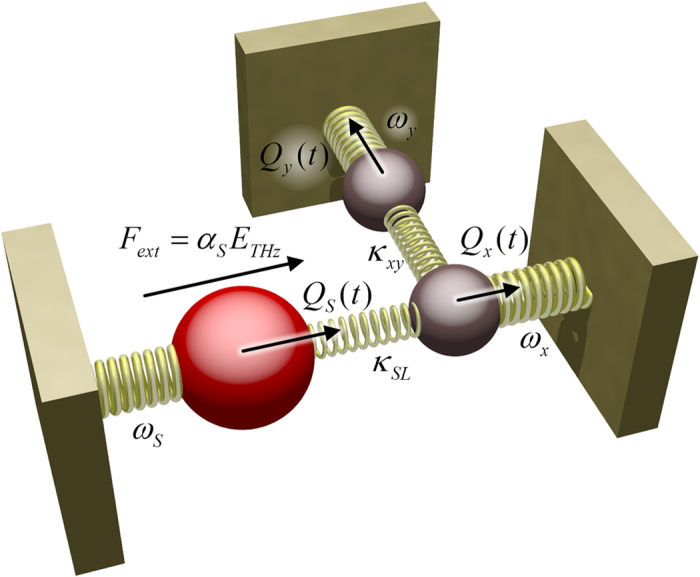
Schematic illustration of the mechanical analog of the coupled oscillator system of the slit resonator and two lattice modes. The slit resonance couples only to the x-lattice mode, and there is coupling between the x- and y-lattice modes.

In a system of two or more resonant modes closely spaced in frequency, where one modes couples to the electromagnetic field (bright mode) and the other modes are dark, but couple to the bright mode, the electromagnetic response of the system can be predicted by the model for coupled mechanical oscillators. In a two-mode system where the damping of the dark mode is much smaller than that of the bright mode, the system behavior closely resembles the classical analog of electromagnetically induced transparency (EIT), with a typical Fano-like^41^ appearance of the spectrum due to the coupling and consequently interference between the two modes. The classical analogy between interacting resonances in a metamaterial and a system of coupled mechanical oscillators is well known^42^,^43^,^44^, where treatments typically have been focused on two coupled oscillators physically located within a distance much smaller than the resonance wavelength. Coupling between two spatially separated oscillators can be treated by introduction of a phase retardation in the coupling coefficient^45^, and this retardation can account for the opposite effect of EIT, namely electromagnetically induced absorption (EIA). Here we show that a coupled oscillator model with a retarded coupling reproduces all important features observed in our experimental results of delocalized lattice resonances interacting with a two-dimensional array of identical resonators. Figure 9 shows a schematic illustration of the coupling between the slit and the x-direction lattice mode, and the coupling between the x- and y-direction lattice modes.

First we establish the relation between the field enhancement in the center of an isolated slit and the motion of a harmonic oscillator driven by an external force. The differential equation for this system is


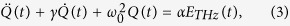


where *γ*, *ω*_0_, α, *E*_*THz*_ is the damping rate, the undamped resonance frequency, the coupling constant to the electric field, and the external field, respectively. At frequency *ω* and *Q(t*) = *q(ω*)exp(−*iωt*), the solution to Eq. (3) is


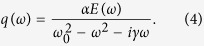


**Figure 10:**
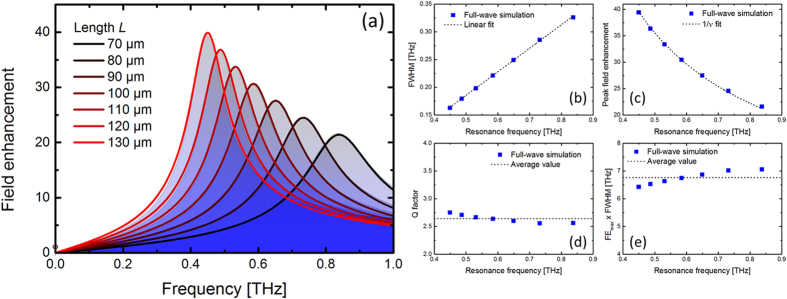
(**a**) Simulation (blue-shaded filled area) of the field enhancement in the center of an isolated slit in a 200-nm gold film on the surface of a silicon substrate, for lengths of the slit *L* = 70–130 μm. The full, red-hued curves are fits with the analytical oscillator model described in the text, using a single polarizability parameter and loss coefficient. (**b**–**e**) Show Full width at half maximum (FWHM) of the resonances, maximum field enhancement (FE_max_), quality factor (Q) and area under the curve (FWHM × FE_max_).

The field enhancement in the slit is a result of the polarization field set up by the external field, which for the harmonic oscillator corresponds to *p(ω*) ∝ |*q(ω*)|. The blue shaded areas in Fig. 10(a) shows full-wave simulations of the field enhancement in an isolated slit in a metal film of 200 nm thickness on a silicon substrate for slit lengths *L* = 70–130 μm and width 1 μm. Figure 10(b) and (c) shows that the full width at half maximum (FWHM) of the resonance peaks scales linearly with frequency, and that the peak field enhancement scales with the inverse of frequency, and thus the Q factor (Fig. 10(d)) and area under the field enhancement curve (Fig. 10(e)) are almost constant. Thus, the damping rate and the coupling between the electric field and the resonance both increase linearly with frequency, *γ* = *γ*′·*ω* and *α* = *α*′·*ω*.

The full curves in red hues in Fig. 10(a) shows |*q(ω*)| for the various slit lengths, using the fitted values for *α*′ and *γ*′ for the *L* = 130 μm case. The perfect agreement between the analytical model and the full-wave simulation results show that the basic physics of the field enhancement is that of a driven harmonic oscillator, and that the amplitude of the oscillator motion is the mechanical equivalent of the observed field enhancement.

For the full analytical model for the coupling between the slit resonance and the lattice modes we assume individual resonance frequencies and damping rates *ω*_*s*_, *ω*_*x*_, *ω*_*y*_, *γ*_*s*_, *γ*_*x*_, *γ*_*y*_ for the slit and lattice modes in the x- and y-direction respectively, and the same frequency dependence of *γ*_*S*_ and *α*_*S*_ as for an isolated slit. The external electric field couples only to the slit mode due to the x-polarization of the field which cannot directly excite the SPP lattice modes. Due to the significant z-component of the electric field at the long edges of the slit, there is a coupling *κ*_*SL*_ between the slit mode and the lattice mode in the x-direction. Due to the finite extent of the slit there will be a component of the SPP field in the y-direction, resulting in a coupling *κ*_*xy*_ to the lattice modes in the y-direction. The coupled motion of the three oscillators can then be described by a linear set of equations


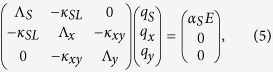


with the solution


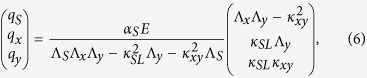


where *q*_*s*_, *q*_*x*_, *q*_*y*_ are the complex amplitudes of the slit and lattice modes, respectively, and 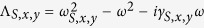. From close inspection of the field patterns from full-wave simulations (e.g. Fig. 7) we find that there is a phase difference between the oscillation of the z-component of the field along the slit edges and the x-lattice mode. This phase difference (approximately 20°) as well as a phase difference of 90° between the x- and y-lattice modes has been used. The frequency-dependent damping rate of the slit mode (*γ*_*s*_ = 0.22·*ω*) is taken from the initial investigation of the isolated slit. The damping of the lattice modes is taken as independent of frequency, *γ*_*x*, *y*_ = 2*π*·0.1 THz. The coupling strengths are |κ_*SL*_| = 5 ps^−2^ and |*κ*_*xy*_| = 2.5 ps^−2^.

**Figure 11:**
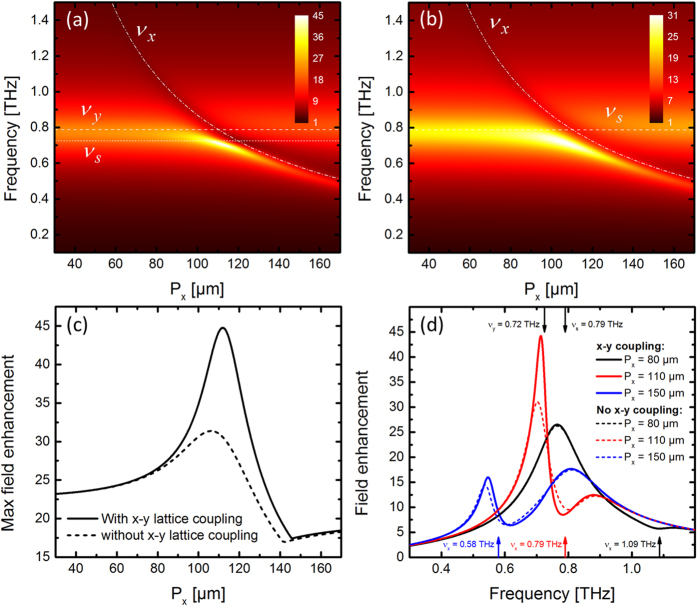
Amplitude of *q*_*s*_ with sweeps of the lattice constant *P*_*x*_ and constant *L* = 75 μm and *P*_*y*_ = 120 μm (**a**) with and (**b**) without coupling to the x-y lattice mode coupling. (**c**) Maximum field enhancement with and without x-y coupling, and (**d**) amplitude spectra for selected *P*_*x*_ values with and without x-y coupling.

Figure 11 offers an overview of the full analytical model, and the delicate influence of the x-y lattice coupling. The contour plots in Fig. 11(a) shows the amplitude of the oscillator |*q*_s_|, for comparison with the experimental and simulation results in Fig. 3. In Fig. 11(b) the x-y lattice coupling has been disabled. The redshift of the slit resonance *v*_*s*_ as the x-lattice modes cross the slit mode is obvious. Figure 11(c) shows the maximum field enhancement for each value of *P*_*x*_, with and without x-y lattice coupling. It is clear that the indirect coupling between the slit and the y-lattice mode leads to larger field enhancement compared than with coupling only to the x-lattice mode. Figure 11(d) shows the field enhancement spectrum for different values of *P*_*x*_, with and without x-y lattice mode coupling. The x-y lattice mode coupling is strongest when the two lattice resonance frequencies are close to each other.

**Figure 12:**
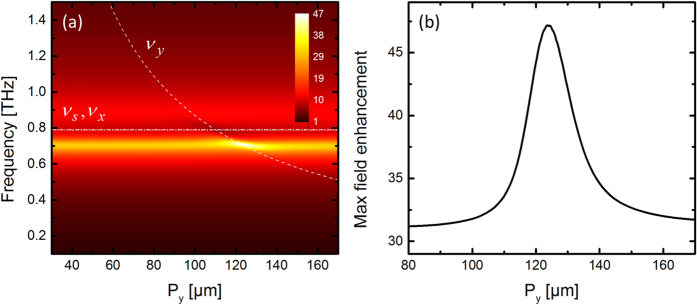
(**a**) Amplitude of *q*_*s*_ for a sweep of the lattice constant *P*_*y*_ and constant *L* = 75 μm and *P*_*x*_ = 110 μm. (**b**) Maximum field enhancement as function of *P*_*x*_.

Similarly, Fig. 12 shows the field enhancement for a sweep of *P*_*y*_. As observed in experiment and simulation, the analytical model predicts that the mode is invisible, except for an additional field enhancement when the dark y-lattice mode crosses the radiative slit mode in the spectral vicinity of the x-lattice mode. These two examples show the close analogy between the coupled system of harmonic oscillators and the observed behavior of the slit array resonance dynamics.

## Conclusion

We have investigated the influence on the coupling between the resonance of micro-slits in a metallic film, arranged in a periodic array so that energy can be coupled to lattice modes of the array. At the resonance frequencies of the lattice modes, the fraction of the transmitted field that couples to SPP waves, and thereby lost in the case of an isolated slit, can be coupled back into the adjacent slits, and thus amplify the field enhancement. We find that careful tuning of the lowest-order lattice modes in both directions of the 2D periodic array to match the resonance frequency of the slit mode leads to a significant additional field enhancement, here approximately 60%, and specifically with a field enhancement increasing from 25 to 40 in the center of a slit with resonance frequency at 0.7 THz. The coupling mechanism between the slit resonance and the lattice modes is precisely reproduced by a model of coupled harmonic oscillators, where the slit mode couples directly to the x-lattice mode giving rise to a Fano-like field enhancement. Indirect coupling to the y-lattice mode further enhances the field inside the slit. Such slit array structures may enable highly sensitive nonlinear studies using high repetition rate spectroscopic systems, with all the advantages in terms of stability noise such systems offer, compared to low-repetition rate systems.

## Methods

### Measured field enhancement

The THz-TDS experiments for determining the field strength inside the micro-slits were performed with a Picometrix T-Ray 4000 fiber-coupled THz spectrometer using focusing lenses with effective focal lengths of 3′′ (76.2 mm), resulting in a spot size at the sample position in the focal plane of approximately 1.5 mm (full width at half maximum, FWHM) at 1 THz. The polarization of the THz beam is parallel to the width of the slit. The THz waveforms have a pulse duration of approximately 0.5 ps and cover a spectral range from 0.1 to 2 THz, and the pulses are recorded in a time window of 320 ps. Each waveform is averaged over 10.000 individual scans with a total acquisition time of 100 s. The transmission of the samples was measured by the standard technique of recording a reference signal *E*_*ref*_(*t*) with an empty beam path, and a sample signal *E*_*sam*_(*t*) at normal incidence through the sample. After Fourier transformation of the signals, the field amplitude transmission coefficient through the film was then calculated as *t(v*) = [(*n*_*Si*_ + 1)^2^/4*n*_*Si*_]·*E*_*sam*_(*v*)/*E*_*ref*_(*v*) to take the Fresnel losses of the substrate of the sample into account. The field enhancement inside the slits is inferred from transmission measurements as |*t(v*)|/*β*, where *t(v*) is the field transmission coefficient through the film obtained in experiment and *β* = (*L* · *w*)/(*P*_*x*_ · *P*_*y*_) is the fill factor^15^. The measured transmission was independent of detailed alignment of the sample in the horizontal and vertical directions due to the large spot size of the THz beam relative to the lattice parameters.

### Fabrication

Samples were fabricated using standard UV photolithography. Each sample had an area of 9 × 9 mm^2^. Table 1 gives an overview of the three series of samples fabricated, with specification of the slit length *L* and lattice parameters *P*_*x*_ and *P*_*y*_, and the associated resonance frequencies. In all cases the slit width (*w*) was 1.5 μm and the thickness of the gold film was 200 nm, thicker than the skin depth in the relevant THz range (70 nm at 1 THz). Figure 1(b) shows a scanning electron microscopy (SEM) image of one of the samples, indicating the various dimensions of interest. The inset in the upper right corner shows a zoom of a section of a slit. The arrays were fabricated on a 525 μm thick high resistivity silicon (HR-Si) substrate.

### Simulations

The full-wave electromagnetic simulations were performed using the time-domain solver of CST Microwave Studio, using periodic boundary conditions with a unit cell size defined by *P*_*x*_ and *P*_*y*_, and with a broadband (0–4 THz) excitation pulse polarized in the x-direction. The frequency-dependent field enhancement was recorded by placing a near-field probe in the center of the slit, while field distributions for specific frequencies were monitored in an xy-plane placed either in the center of the slit or 0.5 μm into the substrate to simulate the field distribution in the slit and of the lattice modes, respectively. To ensure an acceptable resolution of the acquired field distributions, simulations were performed with a custom grid with a fine mesh around the slit edges of 125 nm, while the mesh in between the slits was increased to 1.5 μm to reduce the computational time.

## Additional Information

**How to cite this article**: Klarskov, P. *et al.* Amplification of resonant field enhancement by plasmonic lattice coupling in metallic slit arrays. *Sci. Rep.* **6**, 37738; doi: 10.1038/srep37738 (2016).

**Publisher's note:** Springer Nature remains neutral with regard to jurisdictional claims in published maps and institutional affiliations.

## Figures and Tables

**Table 1:** Overview of sample series, critical dimensions and resonance frequencies.

Series	I	II	III
*L* [μm]	30–105	75	75
*v*_0_ [THz]	1.815–0.537	0.763	0.763
*P*_*x*_ [μm]	70	30–170	110
*v*_*lat*_ (1, 0) [THz]	1.254	2.926–0.516	0.798
*P*_*y*_ [μm]	110	120	80–170
*v*_*lat*_(0, 1) [THz]	0.798	0.731	1.097–0.516
